# Health workforce planning models for older adults in the WHO Eastern Mediterranean Region: a mixed methods systematic review

**DOI:** 10.1186/s12960-026-01077-9

**Published:** 2026-05-15

**Authors:** Salim Ahmed Alshehri, Lucy Simmonds, Angie Shafei, Fany Indriyani, Mohammed Khaled Al-Hanawi, Madhan Balasubramanian

**Affiliations:** 1 Department of Finance, College of Business Administration in Hawtat Bani Tamim, Prince Sattam Bin Abdulaziz University, Al-Kharj, 11942 Saudi Arabia; 2 Flinders Business School | Health Care Management | College of Business Creative Arts Law and Social Sciences, Flinders University, Adelaide SA, Australia; 3 Faculty of Medicine and Health, Leeder Centre for Health Policy, Economics and Data, School of Public Health, The University of Sydney, Sydney, Australia; 4 Department of Health Services and Hospitals Administration, Faculty of Economics and Administration, King Abdulaziz University, Jeddah, Saudi Arabia; 5 Health Economics Research Group, King Abdulaziz University, Jeddah, Saudi Arabia

**Keywords:** Health workforce, Health systems, Planning, WHO Eastern Mediterranean Region, Older adults, Healthcare models, Financial sustainability, Systematic review

## Abstract

**Background/objective:**

As the population across the World Health Organization Eastern Mediterranean Region (EMR) ages, healthcare systems are increasingly strained by the rising burden of non-communicable diseases and the growing demand for chronic geriatric care. Effective health workforce planning is essential to address these evolving needs. However, limited evidence exists on the applicability of workforce planning models tailored specifically to older adults in the EMR. The purpose of this review is to examine current models and strategies in health workforce planning for older adult care in the EMR, with a focus on regionally tailored, financially sustainable, and policy-relevant solutions.

**Methods:**

A mixed methods systematic review following JBI methodology was conducted. Five databases (MEDLINE, Embase, Scopus, CINAHL, and Web of Science) were searched for English-language qualitative, quantitative, and mixed-methods studies examining workforce planning models relevant to older adults’ care in the EMR. The review focused on identifying approaches to workforce supply, demand and needs forecasting, competency development, retention, and financial sustainability in healthcare systems. The data were synthesized using a convergent integrated approach and the included studies were assessed for quality using the QuADS tool.

**Results:**

Among the total 1152 records identified, 11 studies met the inclusion criteria, covering four EMR countries (Iran, Lebanon, Jordan, and Saudi Arabia) across three healthcare settings. The key findings highlighted persistent shortages in geriatric, rehabilitation, and chronic disease specialists; limited use of forecasting models; and insufficient professional development opportunities. Major barriers included health professional migration, poor working conditions, and financial instability. Critical enablers included integrated supply-and-demand forecasting, competency-based training, and retention strategies such as improved remuneration, career progression, and supportive work environments. Financial sustainability was highlighted as a cross-cutting concern.

**Conclusion:**

This review highlights the urgent need for regionally adapted data-driven workforce planning models that integrate forecasting, competency development, and retention strategies to meet the care demands of aging populations in the EMR.

**Supplementary Information:**

The online version contains supplementary material available at 10.1186/s12960-026-01077-9.

## Introduction

A global demographic shift towards population aging is transforming health systems worldwide [1]. The population of people aged 60 and above is gradually rising In the World Health Organization (WHO) Eastern Mediterranean Region (EMR), which consists of Afghanistan, Bahrain, Djibouti, Egypt, Iran, Iraq, Jordan, Kuwait, Lebanon, Libya, Morocco, the Occupied Palestinian Territory, Oman, Pakistan, Qatar, Saudi Arabia, Somalia, Sudan, Syria, Tunisia, the United Arab Emirates, and Yemen [2]. This population shift is associated with an increasing burden of chronic noncommunicable diseases (NCDs) including diabetes, cardiovascular disease, cancer, and chronic respiratory diseases, which disproportionately affect older adults [3, 4]. More than half of all deaths every year and 60% of the healthcare burden in the region are now due to NCDs [4], and Pakistan, Kuwait, and Egypt, which are ranked among the top 10 nations in the world at high risk of diabetes [5].

Although this necessity is increasing, several EMR nations experience severe gaps in specialized health services. Despite these escalating healthcare demands, many EMR countries face critical shortages in specialized services such as geriatrics, rehabilitation, and chronic disease management, which add more pressure to already weak health systems. One of the most significant constraints in addressing this growing need is the limited availability and uneven distribution of healthcare professionals in the EMR. For instance, Lebanon has over 31 physicians per 10 000 people compared to only 6.3 in Morocco [6], and the situation is more severe in high-demand specialty and underserved or rural regions. Emigration to countries such as Egypt, Sudan, and Jourdan also impairs workforce retention due to poor working conditions, the absence of career opportunities, and financial security [7, 8], burnout long working hours, poor infrastructure and professional development [9]. High turnover, low job satisfaction, and the risk of personal safety are added to these problems in politically unstable nations like Yemen, Iraq, Syria, and Afghanistan [10, 11]. Such realities emphasize the importance of integrated health workforce planning in the EMR, based on the demographic, disease, and healthcare utilization data, to develop coordinated, supply- and demand-driven models capable of forecasting future healthcare needs, addressing supply–demand imbalance and determining the appropriate number, distribution, competence, and retention strategies of healthcare professionals required to provide for older adults.

Integrated demand-and-supply forecasting is needed to develop proactive workforce strategies that align future care needs with projected clinical supply. Such a an approach can guide workforce migration, education, and retention strategies, and geographic distribution of workforce, particularly in under-resourced area with high attrition rates [12]. Workforce planning models should adopt competency-based approaches tailored to the EMR context [13]. These should priorities skills in geriatrics and chronic disease management to address the specific needs for older adults.

Workforce planning models must be flexible to respond to evolving health needs, including emerging threats such as pandemics and regional conflicts. In this regard, flexible workforce models capable of helping healthcare systems quickly respond to such demands are key to ensuring the sustainability of the health workforce [14]. This adaptability is especially relevant in the context of the EMR, where health systems encounter unique issues associated with political instability, conflict, and resource limitations.

Beyond workforce planning, health financing is central to sustainability of EMR health systems. The rising costs associated with population aging and the growing burden of chronic disease pose significant threats to financial sustainability [15, 16]. As the need for long-term care, specialty care, and hospital stays increases, healthcare systems are failing to keep up with these rising expenditures, frequently exceeding public funds in play [15]. Many EMR countries allocate less than the recommended 5% of their GDP to health expenditure; consequently, their overdependence on out-of-pocket payments worsens the financial barriers faced by vulnerable groups [17].

In many EMR countries, fragment healthcare financing exacerbates these challenges by creating cost inefficiencies and duplicating resources. Countries experiencing political instability and conflict, notably Syria, Yemen, and Afghanistan, have seen their healthcare infrastructure destroyed and financial resources stretched thin, which in turn has further compromised the financial sustainability of healthcare systems [18]. Deficient innovative financing mechanisms (e.g., public–private partnerships and diverse revenue streams) hamper the ability of the region to secure sustainable healthcare financing [19]. In this context, rehabilitation and long-term care service remain underfunded. Their development should prioritize sustainable system structures capable of responding to the growing needs of older adults in the EMR.

Despite recognition of these challenges, evidence synthesizing healthcare workforce planning for older adults in EMR remains limited. This review addresses this gap by synthesizing workforce planning models and providing regionally relevant insights to inform policy and workforce development for aging population in the EMR. This review focuses on key aspects of workforce planning relevant to financial sustainability in EMR health systems, including supply, demand and needs forecasting, as well as training and retention strategies for older adults care. The findings highlight the need for evidence-based frameworks that respond to region-specific workforce shortage, migration, training gaps, and sustainable financing mechanism. This synthesis identifies gaps in supply-and-demand forecasting, clarifies drivers of competency-based training and retention, and informs targeted policy intervention to strengthen equitable service delivery. This review aims to synthesize current models and strategies in health workforce planning for older adult care in the EMR, with a focus on regionally tailored, financially sustainable, and policy-relevant solutions.

## Methods

This systematic review used a mixed-methods approach, following the *Joanna Briggs Institute Reviewer’s Manual* for a mixed-methods systematic review [20]. The manuscript was prepared according to PRISMA (Preferred Reporting Items for Systematic reviews and Meta-Analyses) guidelines [21]. Specifically, this review synthesizes both qualitative and quantitative research with a convergent integrated approach, thus facilitating the simultaneous preparation of data and analysis from mixed-methods study designs [22]. The data synthesis was conducted narratively to present the findings in a comprehensive manner that highlights trends and facilitates drawing meaningful conclusions to strengthen healthcare workforce planning in the EMR. The systematic review was registered on the International Platform of Registered Systematic Reviews and Meta-analysis Protocols (INPLASY) under ID INPLASY202470013 on July 5, 2024.

### Search strategy

The search strategy was prepared in consultation with a librarian at Flinders University. A comprehensive search protocol was created to identify studies that focus on models, strategies, and interventions for health workforce planning for older people in the EMR. The literature search for this review was performed in five databases: Medline, Embase, Scopus, CINAHL, and Web of Science.

The following keywords and Medical Subject Headings (MeSH) were used in the search strategy according to our review objective: “health workforce planning,” “older adults,” “Eastern Mediterranean Region,” and “healthcare workforce strategies.” Studies published between 1997 and 2024 were included. Duplicate records were identified and excluded using Covidence software, and the remaining studies proceeded through the eligibility screening process described below. The complete search strategy, including an example from MEDLINE with the corresponding numbers of results, is provided in Table 1.

**Table 1:** Ovid MEDLINE search strategy

	Search	Query	Records Retrieved
Theme 1: Health professionals	1	Allied Health Personnel/ or Allied Health Occupations/ or Anesthetists/ or Audiologists/ or Community Health Workers/ or Dental Auxiliaries/ or Dental Staff/ or Dentists/ or Emergency Medical Technicians/ or Health Occupations/ or Health Workforce/ or Home Health Aides/ or Infection Control Practitioners/ or Medical Laboratory Personnel/ or exp Medical Staff/ or Nurses/ or Nursing Assistants/ or Nursing Staff/ or Nutritionists/ or Occupational Therapists/ or Operating Room Technicians/ or Optometrists/ or Pharmacists/ or Pharmacy Technicians/ or Physical Therapy Assistants/ or exp Physician Assistants/ or Physicians/ or Psychotherapists/ or Surgeons/	317,963
	2	(acupuncturist* An?esthetists or clinician* or (dental adj3 assistant*) or (dental adj3 auxiliar*) or (dental adj3 hygienist*) or dentist* or denturist* or dieti?ian* or doctor* or (emergency medical adj4 technician*) or healer* or ("home health*" adj4 aide*) or (lab* adj3 personnel*) or (lab* adj3 worker*) or (lactation adj3 consultant*) or masseuse* or midwife or midwives or naturopath* or nurs* or nutritionist* or optician* or optometrist* or orthoptist* or osteopath* or paramedic or paramedic* or physician* or physiotherapist* or podiatrist* or prosthetist* or psychotherapist* or radiographer* or sonographer* or (health adj3 workforce*) or (healthcare adj3 labo?r) or (healthcare adj3 labor) or (healthcare adj3 staff*) or (healthcare adj3 manpower) or (health adj3 professional*) or (health adj3 worker*) or (human resource* adj3 health*) or (dental adj3 staff*) or (public health* adj4 workforce*) or (nurs* adj3 staff*) or (medical adj3 staff*) or (pharmac* adj3 technician*) or (physical therapy adj4 assistant*) or optometrist* or (occupational adj3 therapist*) or pharmacist* or technician* or (physical therapy adj4 assistant*) or (physician* adj3 assistant*) or physician* or psychotherapist* or (health* adj3 workforce) or (health* adj3 staff*) or (health* adj3 labo?r*) or (health* adj3 employ*) or (health* adj3 crew*) or (health* adj3 personnel*) or (health* adj3 manpower) or (health* adj3 worker*) or (health* adj3 professional*) or ("Allied Health" adj4 Personnel*) or ("Allied Health" adj4 Occupation*) or Anesthetist* or Audiologist* or (Community adj4 "Health* Worker*") or (Dental adj3 Auxiliar*) or (Dental adj3 Staff*) or Dentist* or (Emergency adj4 Medical Technician*) or (Health* adj3 Occupation*) or (Health* adj3 Workforce) or (Home adj4 "Health* Aide*") or (Infection Control adj4 Practitioner*) or ("Medical Laboratory" adj4 Personnel*) or Nurs* or (Nurs* adj3 Assistant*) or (Nurs* adj3 Staff*) or Nutritionist* or (Occupation* adj3 Therapist*) or ("Operating Room*" adj4 Technician*) or Optometrist* or Pharmacist* or (Pharmac* adj3 Technician*) or (Physical Therapy adj4 Assistant*).tw,kf	1,834,166
	3	1 or 2	1,954,933
Theme 2: Health population	4	"Aged, 80 and over"/ or Aged/ or Health Services for the Aged/ or Dental Care for Aged/	3,541,968
	5	((Older adj3 (adult or individual or person* or people or patient* or population* or m?n or wom?n)) or Elder* or Senior* or Geriatric* or (age* adj3 ("60" or "65" or "70" or "75" or "80" or "85")) or (Elder* adj3 population) or (Elder* adj3 people) or (Elder* adj3 patient*) or (Elder* adj3 population)).tw,kf	809,922
	6	4 or 5	3,857,682
Theme 3: Health workforce planning models	7	health care costs/ or direct service costs/ or employer health costs/ or hospital costs/ or health expenditures/ or capital expenditures/ or Healthcare Financing/ or "Health Services Needs and Demand"/	136,631
	8	((Physicians adj3 demand) or ("health* staff*" adj4 demand) or ("health* labo?r*" adj4 demand) or ("health* workforce" adj4 demand) or ("health* employ*" adj4 demand) or ("health* crew*" adj4 demand) or ("health* workforce" adj4 plan*) or ("health* capacity" adj4 plan*) or (health* adj4 "Economic* model*") or ("financ* model*" adj4 health*) or (health* adj3 insurance) or ("national* health*" adj4 insurance) or ("cost* effective*" adj4 health*) or ("Health* insurance" adj4 cost*) or ("econometric* model*" adj4 health*) or ("health* demand" adj4 analysi*) or (future adj3 health*) or ("Health* Workforce" adj4 Utilization) or ("Health* Demand" adj4 Forecast*) or ("Health* Supply" adj4 Projection*) or ("Health* Gap*" adj4 Analysi*) or ("Health* Strateg*" adj4 Develop*) or ("Health* Workforce" adj5 "Projection* Model*") or (health* adj4 "simulation model*") or (Health* adj4 "Needs-based Model*") or (Health* adj4 "Benchmark* Model*") or ("Health* staff*" adj4 expend*) or ("Health* labo?r*" adj4 expend*) or ("Health* member*" adj4 expend*) or ("Health* employ*" adj4 expend*) or ("Health* crew*" adj4 expend*) or ("Health* personnel*" adj4 expend*) or ("Medical workforce" adj4 cost*) or ("Medical labo?r*" adj4 cost*) or ("Medical member*" adj4 cost*) or ("Medical employ*" adj4 cost*) or ("Medical crew*" adj4 cost*) or ("Health* staff*" adj4 expense*) or ("Health* labo?r*" adj4 expense*) or ("Health* member*" adj4 expense*) or ("Health* employ*" adj4 expense*) or ("Health* crew*" adj4 expense*) or ("Health* personnel*" adj4 expense*) or ("Health* staff*" adj4 budget*) or ("Health* labo?r*" adj4 budget*) or ("Health* member*" adj4 budget*) or ("Health* employ*" adj4 budget*) or ("Health* crew*" adj4 budget*) or ("Medical staff*" adj4 cost*) or ("Medical labo?r*" adj4 cost*) or ("Medical member*" adj4 cost*) or ("Medical employ*" adj4 cost*) or ("Medical crew*" adj4 cost*) or ("Cost* health*" adj4 staff*) or ("Cost* health*" adj4 labo?r*) or ("Cost* health*" adj4 member*) or ("Cost* health*" adj4 employ*) or ("Cost* health*" adj4 crew*) or ("Cost* health*" adj4 provider*) or ("Cost* health*" adj4 workforce) or ("Health* staff*" adj4 financ*) or ("Health* labo?r*" adj4 financ*) or ("Health* member*" adj4 financ*) or ("Health* employ*" adj4 financ*) or ("Health* crew*" adj4 financ*) or ("Health* workforce" adj4 financ*) or ("Cost* clinical" adj4 staff*) or ("Cost* clinical" adj4 labo?r*) or ("Cost* clinical" adj4 employ*) or ("Cost* clinical" adj4 crew*) or ("Cost* clinical" adj4 workforce) or ("Health* staff*" adj4 fund*) or ("Health* labo?r*" adj4 fund*) or ("Health* employ*" adj4 fund*) or ("Health* crew*" adj4 fund*) or ("Health* workforce" adj4 fund*) or ("Medical staff*" adj4 expense*) or ("Medical labo?r*" adj4 expense*) or ("Medical member*" adj4 expense*) or ("Medical employ*" adj4 expense*) or ("Medical crew*" adj4 expense*) or ("Health* staff*" adj4 shortag*) or ("Health* labo?r*" adj4 shortag*) or ("Health* workforce" adj4 shortag*) or ("Health* employ*" adj4 shortag*) or ("Health* crew*" adj4 shortag*) or ("Health* staff*" adj4 deficit*) or ("Health* labo?r*" adj4 deficit*) or ("Health* workforce" adj4 deficit*) or ("Health* employ*" adj4 deficit*) or ("Health* crew*" adj4 deficit*) or ("Health* worker*" adj4 deficit*) or ("Health* staff*" adj4 shortfall) or ("Health* labo?r*" adj4 shortfall) or ("Health* workforce" adj4 shortfall) or ("Health* employ*" adj4 shortfall) or ("Health* crew*" adj4 shortfall) or ("Health* staff*" adj4 gap*) or ("Health* labo?r*" adj4 gap*) or ("Health* workforce" adj4 gap*) or ("Health* employ*" adj4 gap*) or ("Health* crew*" adj4 gap*) or ("Health* staff*" adj4 insufficien*) or ("Health* labo?r*" adj4 insufficien*) or ("Health* workforce" adj4 insufficien*) or ("Health* employ*" adj4 insufficien*) or ("Health* crew*" adj4 insufficien*) or ("Health* staff*" adj4 scarcit*) or ("Health* labo?r*" adj4 scarcit*) or ("Health* workforce" adj4 scarcit*) or ("Health* employ*" adj4 scarcit*) or ("Health* crew*" adj4 scarcit*) or ("Financ* sustainab*" adj4 health*) or ("Financ* stab*" adj4 health*) or ("Econom* sustainab*" adj4 health*) or ("Sustainab* financ*" adj4 health*) or ("Econom* stab*" adj4 health*) or (Financ* adj3 health*) or ("Financ* plan*" adj4 health*) or ("Cost manag*" adj4 health*) or ("Financ* manag*" adj4 health*) or ("Sustainab* econom*" adj4 health*) or ("Econom* viability" adj4 health*) or ("health* staff*" adj4 train*) or ("health* labo?r*" adj4 train*) or ("health* workforce" adj4 train*) or ("health* employ*" adj4 train*) or ("health* crew*" adj4 train*) or ("health* staff*" adj4 retention*) or ("health* labo?r*" adj4 retention*) or ("health* workforce" adj4 retention*) or ("health* employ*" adj4 retention*) or ("health* crew*" adj4 retention*) or ("health* staff*" adj4 strateg*) or ("health* labo?r*" adj4 strateg*) or ("health* workforce" adj4 strateg*) or ("health* employ*" adj4 strateg*) or ("health* crew*" adj4 strateg*)).tw,kf	111,073
	9	7 or 8	237,599
Theme 4: Mediterranean countries	10	Mediterranean Region/ or middle east/ or egypt/ or libya/ or morocco/ or tunisia/ or djibouti/ or somalia/ or sudan/ or bahrain/ or iran/ or iraq/ or jordan/ or kuwait/ or lebanon/ or oman/ or qatar/ or saudi arabia/ or syria/ or united arab emirates/ or yemen/ or afghanistan/ or pakistan/ or Palestine/	167,597
	11	(Afghanistan or Kabul or Kandahar or Bahrain or Manama or Muharraq or Djibouti or "Ali Sabieh" or Egypt or Cairo or Alexandria or Giza or iran or Tehran or Mashhad or Iraq or Mosul or Basra or Jordan or Zarqa or Irbid or Kuwait or "Al Ahmadi" or Hawalli or Lebanon or Beirut or Tripoli or Libya or Benghazi or Morocco or Rabat or Casablanca or Marrakesh or oman or Muscat Salalah or Pakistan or Islamabad or Karachi or Lahore or Palestine or Gaza or Nablus or Ramallah or Qatar or "Al?Rayyan" or "Saudi Arabia" or "kingdom of Saudi Arabia" or "KSA" or Riyadh or Jeddah or Mecca or Medina or Dammam or Somalia or Mogadishu or Hargeisa or Sudan or Khartoum or Omdurman or "Port Sudan" or Syria or Damascus or Aleppo or Homs or Tunisia or Tunis or Sfax or Sousse or "United Arab Emirates" or Emirates or "Abu?Dhabi" or Dubai or Sharjah or "Al?Ain" or Ajman or Yemen or Sana'a or Aden or Taiz).tw,kf	229,582
	12	11 or 12	283,710
	14	3 and 6 and 9 and 12	169

### Eligibility criteria

This review included original research studies employing qualitative, quantitative, or mixed-methods designs that examined health workforce planning models, strategies, or interventions pertinent to the care of older adults in the EMR. Studies were eligible for inclusion if they reported on workforce challenges, training and retention approaches, or demand-and-supply forecasting models related to healthcare professionals providing services to older populations within the EMR.

We excluded case reports, opinion pieces, study protocols, and studies not published in English. Furthermore, studies not explicitly addressing workforce planning models, strategies, or interventions designed for older adults in the EMR were excluded. Such selection ensured that the studies included in the review were pertinent to answering its primary question.

### Study selection

The study selection was performed through a two-step process involving Covidence [23]. This online tool helps manage the systematic review process by guiding users through each step, such as screening titles and abstracts, full-text review, data extraction, and quality assessment, and automatically moving studies to the next stage when finished [23]. Two independent reviewers (SA and FI) screened the titles and abstracts of all retrieved papers for relevance, applying the eligibility criteria to each study. Differences of opinion between reviewers on whether to include or exclude studies were resolved by discussion. If disagreement persisted between the two reviewers, a third reviewer (MB) was involved to reach a consensus.

The full texts of these eligible studies were subsequently retrieved and assessed for inclusion by the same two independent reviewers. The inclusion decisions were documented along with the reasons for excluding studies according to pre-specified eligibility criteria. This transparency helped ensure the selection process was rigorous and reproducible.

### Data extraction and synthesis

Data extraction was based on the mixed-methods systematic review process as established by the JBI [20] using a pre-piloted form in Microsoft Excel. Independent reviewers extracted relevant data from the included studies. Information relating to the author and year of publication, country of origin, study design, sample size, aims, healthcare setting, and population(s) was captured on a data extraction form developed based on the JBI qualitative data extraction tool [24]. The form also included fields for information on the results of the studies pertaining to workforce planning for older adults classified according to the following domains: barriers, facilitators, training needs, retention strategies, and gaps in workforce planning.

### Data synthesis and integration

The convergent integrated approach as recommended in the JBI methodology for mixed-methods reviews was used for the synthesis of qualitative and quantitative data extracted from the included studies. This enabled the simultaneous analysis of both data types by grouping and categorizing the data regarding themes and findings. The findings were collated into a verbal synthesis, with the main trends and similarities and differences between studies described. To construct a “line of argument,” synthesized results were linked to describe broad themes and to connect challenges and strategies for workforce planning across the EMR [24].

### Quality assessment

Two independent reviewers used the Quality Assessment with Diverse Studies (QuADS) tool to assess the quality of qualitative studies [25]. Each study was assessed against 10 quality criteria and rated as “yes,” “no,” or “partially adhering,” depending on its compliance. This process was standardized so that the methodological quality of all included studies was consistently evaluated.

## Results

**Fig. 1:**
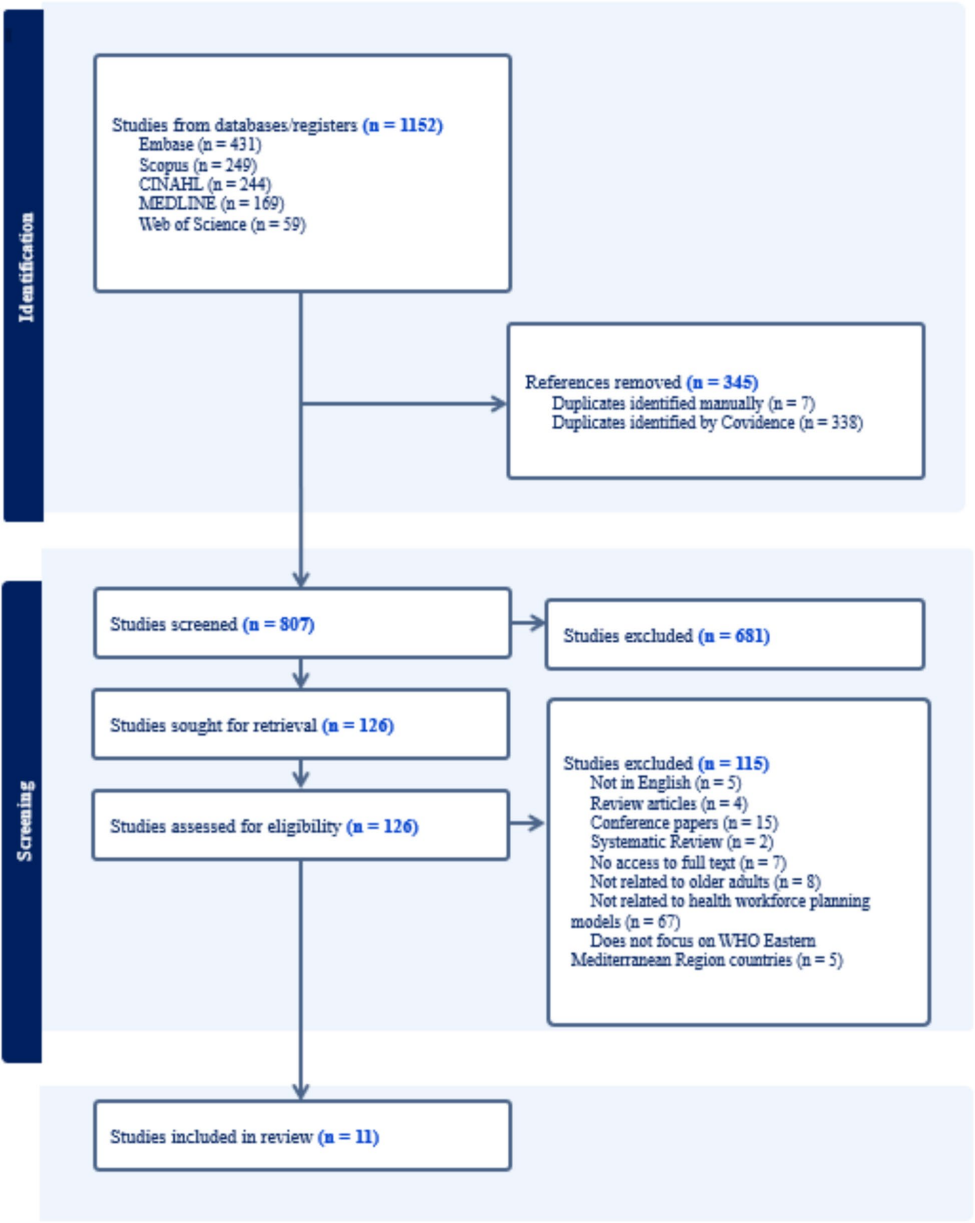
(PRISMA) flow-chart diagram

The search across five databases resulted in 1152 publications, with 345 duplicates removed. During the screening stage, 807 articles were assessed based on their titles and abstracts, resulting in 126 publications being selected for full-text review. Of these studies, 115 were excluded, leaving 11 finally included for the review. The details of the study selection process and reasons for exclusion are presented in the PRISMA flow diagram in Fig. 1.

**Fig. 2:**
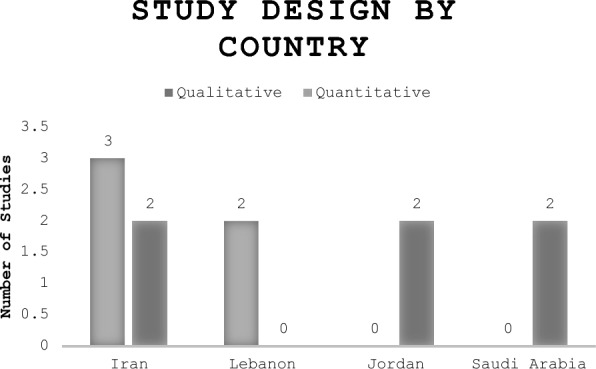
Study design by country

All studies included in this systematic review were conducted in the EMR and consisted of qualitative (*n* = 6) and quantitative (*n* = 5) designs (Fig. 2). The studies were conducted in four countries (Iran, Lebanon, Saudi Arabia, and Jordan) and covered three healthcare settings: primary healthcare centers, elderly care homes, and teaching hospitals (see Table 2).

**Table 2:** Main features of included studies

Study ID	Author-year	Country	Study design	Study aims	Sample size	Healthcare setting
1	Moghadasi, 2022	Iran	Qualitative	Investigate barriers to the usage of the Integrated Aging Program and propose solutions to improve service utilization among older adults	29 older adults and 18 healthcare employees	Primary healthcare centers in Ghaemshahr, Iran
2	Yektatalab, 2012	Iran	Qualitative	Explore caregivers’ experiences and identify key aspects of care for Alzheimer’s patients	14 caregivers (4, 10) working in elderly care homes	Elderly care homes, Shiraz, Iran
3	Gharibian Adra, 2015	Lebanon	Qualitative	Understand and define quality of life from the perspectives of residents, staff, and family caregivers	39 (8, 11, 20)	Care homes for the elderly in Beirut, Lebanon
4	Nojomi, M., 2023	Iran	Qualitative	Identify factors affecting medical students’ attitudes toward older adult care and propose strategies for improvement	27 general medical internship students	Teaching hospitals and medical universities in Iran
5	Al Shammari, 1997	Saudi Arabia	Quantitative	Assess physicians' opinions on the need for home visits, the types of illnesses requiring home visits, and the personnel involved	634 (396 PHC doctors, 238 hospital doctors)	Primary Healthcare Centers and Hospitals in Saudi Arabia
6	Shaheen, 2019	Jordan	Quantitative	Assess the quality of care for older adults and determine the predictors of quality based on work environment and nurse competence	500 nurses providing care for older adults	Hospitals, Health Centers in Jordan
7	Wasfi, 2024	Saudi Arabia	Quantitative	Evaluate the rehabilitation workforce supply, demand, and need across regions and investigate factors influencing workforce availability	32 million (total population)	Ministry of Health facilities, regional health centers across Saudi Arabia
8	Ahmadi, 2015	Iran	Quantitative	Investigate the preparedness of hospitals to cater to elderly patients based on the WHO’s age-friendly hospital framework, focussing on information and training, healthcare management systems, and physical environment	26 public hospitals	Public hospitals in Tehran, Iran
9	Alameddine, 2015	Lebanon	Qualitative	Understand the factors affecting HRH recruitment and retention and provide recommendations for improvement	22 key informants from the Primary Healthcare (PHC) sector	Primary healthcare centers in Lebanon (urban, semi-urban, and rural settings)
10	Nasrabadi, 2021	Iran	Qualitative	To explore the nurses’ experience of moral distress in the long-term care of older adults via a phenomenological study	9 ICU nurses (4 males and 5 females) for older adults	intensive care units (ICUs) in hospitals affiliated with Tehran University of Medical Sciences, Iran
11	El‐Hneiti, 2018	Jordan	Quantitative	To identify sources of stress, examine job satisfaction, work conditions, competence, and skill development, and determine key predictors of stress among nurses in elderly care settings	Nurses providing care for older adults (65 + years) in acute care settings across public hospitals, private hospitals, and healthcare centers in Amman	Public hospitals, Private hospitals, and Healthcare centers in Amman, Jordan

### Study characteristics

**Fig. 3:**
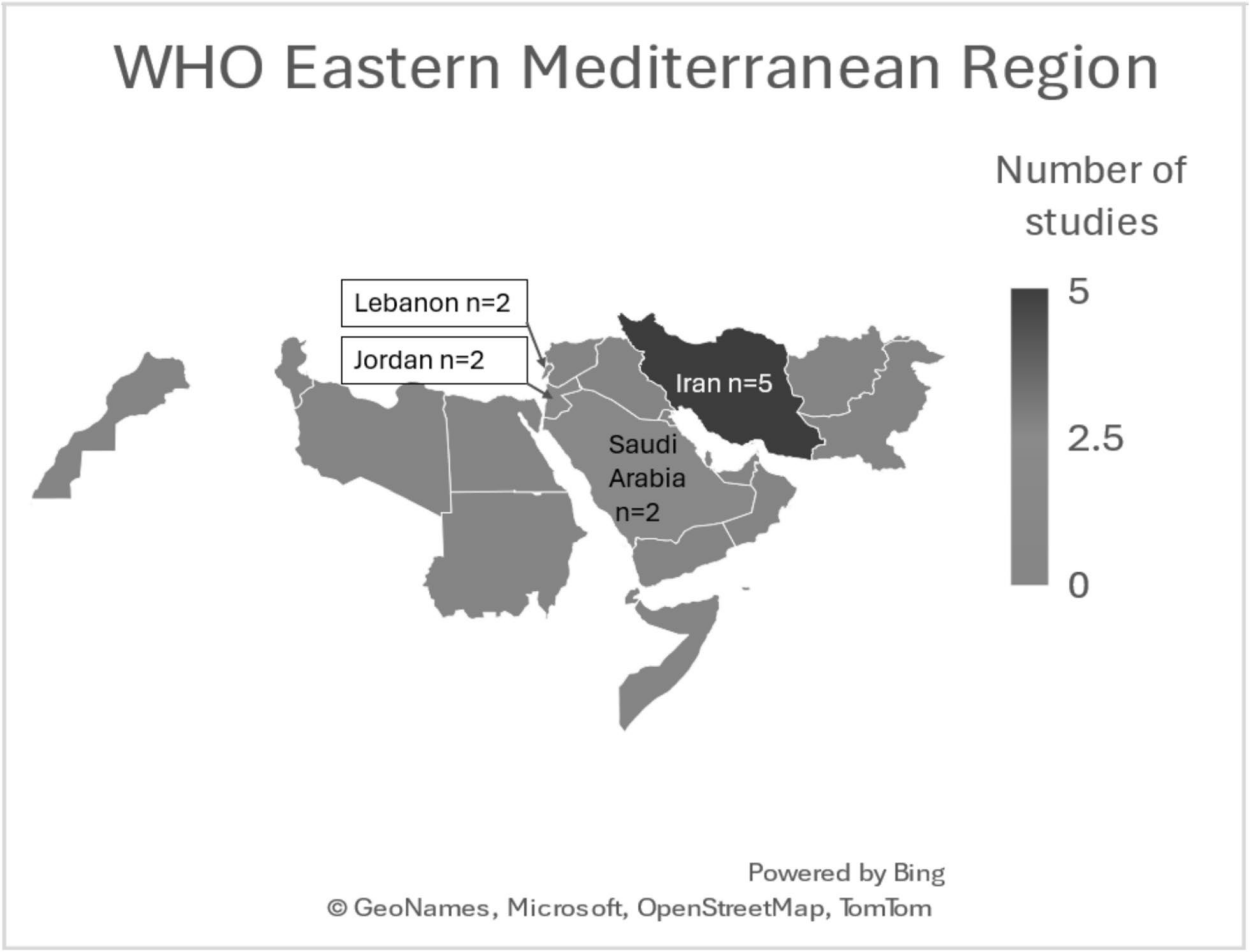
Number of studies in the WHO Eastern Mediterranean Region

Among the 11 studies, four countries in the WHO Eastern Mediterranean Region were represented: 5 in Iran, 2 in Lebanon, 2 in Jordan, and 2 in Saudi Arabia (Fig. 3). The sample sizes varied among the studies. Quantitative studies had the largest samples; for example, the study by Wasfi et al. [26] in Saudi Arabia used population-level data, covering 32 million people, while the study of Al Shammari [27] included 634 healthcare professionals. In contrast, a smaller quantitative study by Ahmadi et al. [28] involved 26 hospitals. Qualitative studies tended to have smaller and more focussed samples. The largest qualitative sample was in the study of Adra et al. [29], which included 39 participants (residents, family caregivers, and staff) in elderly care homes in Lebanon, whereas the smallest was in the study of Nikbakht Nasrabadi et al. [30], which explored moral distress among 9 intensive care unit nurses caring for older adults in Iran. These studies uniformly aimed to understand various elements that affect provider workforce planning, particularly in the context of older adults in the EMR (see Table 2).

### Key aims and findings of the included studies

The main objective of these 11 studies was to investigate various components of healthcare workforce planning for older adults in the WHO EMR, including workforce supply-and-demand forecasting along with training and retention strategies, in response to the increasing demand for healthcare services. For example, Moghadasi et al. [31] conducted a study in Ghaemshahr, Iran, to explore barriers to implementing an integrated aging program and proposed solutions to improve service utilization among older adults. Yektatalab et al. [32] explored caregivers’ experiences and identified the challenges related to workforce planning in elderly care homes in Shiraz, Iran. These studies provide insight into the dynamics of caregiving in institutional settings, focussing on the caregiver burden, staff limitations, and quality of care provided to older adults. Both studies critically examine whether the existing workforce is adequately aligned with the needs of older adults, an essential consideration given the region’s demographic transition and rising demand for elderly care services (see Table 2).

### Healthcare settings

The studies’ settings ranged from primary healthcare centers to aged care facilities to hospitals [29]. For example, a study in Iran investigated caregivers’ experiences in elderly care homes [32]. The variety of settings encompassed in these studies further emphasizes the complex pressures encountered by the healthcare workforce. El-Hneiti et al. [33] note that nurses across various settings in Amman, Jordan, faced different levels of job satisfaction and stress, with the acute care environment placing greater emotional and workload pressure on the workforce (see Table 2).

### Workforce demand and supply

**Fig. 4:**
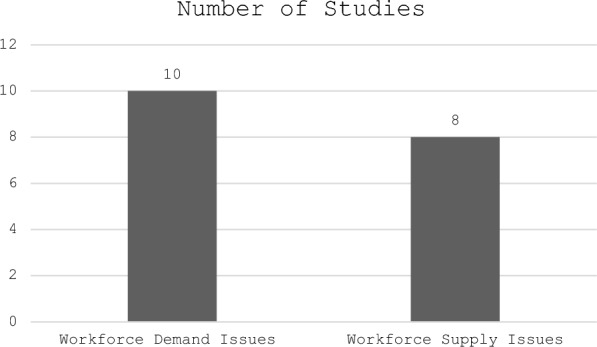
Number of studies mentioned issues regarding the workforce demand and supply of healthcare workers

Several key studies in the WHO EMR have highlighted the need to predict the demand and supply of the workforce, primarily due to the increasing prevalence of chronic diseases in aging societies [26]. An early quantitative study by Al Shammari [27] exploring physicians’ views on the necessity of home healthcare services to meet the growing demands for care among older people in Saudi Arabia. A common recommendation from other studies was the need for integrated and evidence-based workforce planning models to estimate future healthcare needs and shortages of healthcare professionals in specific disciplines such as geriatric medicine and chronic disease management [7, 26, 28] (see Table 3; Fig. 4).

**Table 3:** Health workforce planning characteristics

Study ID	Author-Year	Objective	Implementation challenges	Types of workforces	Intervention/model	Key Health workforce planning focus	Workforce supply (Findings)	Workforce demand (Findings)	Forecasting and projections (Findings)	Training and Retention Strategies (Findings)	Financial Sustainability (Findings)	Key Outcomes
1	Moghadasi, 2022	Explore why older adults in Iran do not use the public health services of the Integrated Aging Program	Lack of awareness, physical and social barriers, inefficiency of healthcare services, and dissatisfaction with available services	general practitioners, primary healthcare providers	Integrated aging program	Barriers to health service access for older adults in Iran	Lack of awareness among patients	High demand for healthcare services but low utilization	No projections	Lack of staff, undertrained health professionals	Poor healthcare insurance coverage	Participants report barriers such as lack of knowledge, high costs, and poor infrastructure
2	Yektatalab, 2012	To explore Iranian caregivers’ perceptions of caring for Alzheimer’s patients in elderly care homes	Limited cultural knowledge of Alzheimer's care, lack of formal education for caregivers, and shortage of trained professionals	Caregivers, head nurses, and supervisors	Routine care for Alzheimer’s patients in elderly care homes	Focus on the physical, psychological, social, and spiritual care of Alzheimer’s patients	Caregivers mostly have no formal education in gerontology; informal caregivers with personal experience	Caregivers report a lack of formal training, a need for professional education, and practical experience	No projections	Lack of specialized training; informal caregivers with inadequate knowledge	N/A	Care mainly focussed on daily physical routines with little emphasis on cognitive or psychological care
3	Gharibian Adra, 2015	Explore the perspectives of quality of life for older residents, care staff, and family caregivers in Lebanese care homes	Limited gerontological education for care staff, understaffing, lack of standardized care models, and cultural factors impacting care delivery	Care home staff and family caregivers	Exploration of the meaning of quality of life from resident, staff, and family perspectives	The importance of personalized, relationship-centred care is a need for culturally relevant approaches	Lack of gerontological training for staff in Lebanon; untrained physicians, nurses, and therapists	Specialist, relationship-focussed care for elderly residents is needed, but it is underprovided due to workforce shortages	No projections;	Lack of specialized training in gerontology and geriatric rehabilitation practices	Lebanese care homes are mostly private with limited public support, relying on government funding, which challenges financial sustainability	Four key categories were identified for quality of life: family connectedness, engaging activities, relationships, and spirituality
4	Nojomi, M., 2023	Explore medical students’ attitudes towards caring for older adults	Negative attitudes (ageism), lack of inter-professional education, inadequate communication skills, and insufficient infrastructure	Medical students, educators	Exploration of attitudes toward older adults' care	Focus on medical students' training gaps for older adult care	Geriatric care suffers from inadequate training, specialist shortages,	Aging populations demand more geriatric specialists	No projections	Need for interprofessional education, communication skills training, and experiential learning for medical students	Limited resources for older adult care; lack of geriatric specialists	Key issues: Ageism, communication barriers, lack of interdisciplinary training, infrastructure limitations
5	Al Shammari, 1997	Examine the perception of physicians on the nature of illnesses requiring home visits, and the type and responsibilities of health personnel needed	Limited home health care infrastructure, variability in physician opinions on home visit requirements, lack of interdisciplinary cooperation	Primary health care doctors, hospital doctors, nurses, physiotherapists, occupational therapists, social workers	Study on physicians' perspectives on home visits to elderly patients	Focus on primary healthcare (PHC) and hospital doctors' perceptions of home visits	Shortage of geriatric specialists, uneven workforce distribution. PHC doctors show greater willingness for home visits, highlighting the need for workforce expansion, training, and logistical support	The aging population increases the demand for geriatric specialists, home healthcare nurses, and physiotherapists, requiring more trained professionals in PHC centers to enhance accessibility and reduce hospital reliance	There are no projections. Recommends support, no workforce estimates	Limited home healthcare training. Enhanced geriatric training, professional development, incentives, and logistical support are key to retaining healthcare providers in elderly home care	Expand elderly home care in Saudi Arabia by integrating PHC teams and prioritizing home visits as a cost-effective alternative to hospitalization	workforce shortages, rising elderly care demand, training gaps, home healthcare needs, and financial sustainability challenges requiring policy and system improvements​
6	Shaheen, 2019	To describe the quality of care for older adults in Jordan and examine predictors of care quality	Insufficient staffing, high occupational stress, lack of employee development, inadequate work climate, limited time for direct patient care	Nurses in hospitals and health centers	Quality of care for older adults	Occupational stress, work climate, employee development, Expanding geriatric education in nursing programs, nurse retention, team-based care models	80.4% of Nurses reported insufficient staffing and organizational changes	Rising demand from an aging population by 2030 is straining care systems already challenged by low staffing	Projections present that demand is expected to rise with the aging population, reaching 782,000 individuals aged 60 and over in Jordan by 2030	Need for improved training, employee development	Low wages and limited advancement opportunities in Jordan have contributed to the migration of experienced nurses to higher-paying positions in Gulf countries	Perceived quality of care was directly related to nurse competence
7	Wasfi, 2024	Assess the supply and demand for rehabilitation health workers (physiotherapists and occupational therapists) in Saudi Arabia	Disparity in workforce distribution across regions, limited data on non-stroke rehabilitation workforce, challenges in aligning workforce supply with rehabilitation needs	Physiotherapists, occupational therapists, healthcare policymakers	Workforce supply and demand for rehabilitation Health workers	Rehabilitation workforce planning should ensure regional equity, expand occupational therapy, use data-driven strategies, align with Vision 2030, and broaden workforce monitoring to improve accessibility and effectively meet rehabilitation needs	Rehabilitation needs index from 0.144 (Najran) to 0.212 (Aseer)	rising rehabilitation demand stems from an aging population and chronic diseases, yet workforce distribution does not align with needs, requiring better planning, regional equity, and increased occupational therapists	No projections	N/A	N/A	The rehabilitation workforce exceeds regional averages but remains below high-income countries. Its uneven distribution, occupational therapist shortages, and rising demand require better workforce planning and targeted recruitment
8	Ahmadi, 2015	To assess the age-friendliness of hospitals in Tehran, Iran, for the growing elderly population	Lack of specialized services for the elderly, limited training for staff in geriatrics, no designated care coordinators for elderly patients, and absence of senior-friendly hospital policies	Hospital staff (nurses, physicians, administrative staff), healthcare administrators	Assess age-friendliness of hospitals	Poor training in geriatrics; no protocols for elderly screening	Age-friendly hospitals face a critical shortage of geriatric physicians, a lack of home health services	The elderly population in Tehran is projected to grow significantly by 2050. As the population ages, hospitals will need to increase their workforce capacity to meet the demands of geriatric care	Projections present. Elderly to reach 25% by 2050	Limited training in geriatrics, no geriatrics-specific care coordinators	N/A	Hospitals lack geriatric training, elderly care infrastructure, screening protocols, and priority services, requiring policy reforms and workforce development​
9	Alameddine, 2015	Explore the recruitment and retention challenges of human resources for health (HRH) in Lebanon's primary healthcare centers (PHCC)	Shortage of healthcare workers, especially in rural areas, lack of specialized HRH in mental health and geriatrics, gender imbalance, and lack of incentives for working in the PHCC	PHC staff (nurses, physicians, social workers, administrators)	Recruitment and retention of health professionals in PHC centers	HRH shortages in rural areas, gender imbalance in HRH, and insufficient family physicians	Staff shortages, gender imbalances, a lack of family physicians, and insufficient geriatric and mental health specialists are negatively impacting primary healthcare services	There is a strong demand for family medicine specialists, mental health, and geriatric care professionals	Projections present. By 2050, 26% of Lebanon’s population will be 65 + , driving urgent demand for expanded geriatric services	Low wages, lack of professional development, and heavy workloads were identified as barriers to retention	Low wages, limited professional development, and poor financial support undermine staff retention and threaten the financial sustainability of PHC services in Lebanon	Lack of qualified HRH in rural and remote areas; absence of mental health and geriatric services
10	Nasrabadi, 2021	To explore ICU nurses' moral distress in long-term older adult care using a phenomenological approach	Nurses face staffing shortages, high workloads, inadequate training, institutional barriers, and emotional burdens	Nurses (CCNs)	Advocacy, training, peer support, teamwork, workload management, institutional support, resilience programs, and ethical guidance	Staffing, training, retention, teamwork, workload management, ethical support, mental health, and policy development for workforce planning	Staffing shortages, high workload,	Demand is increasing for skilled nurses, ethical training, interdisciplinary teamwork, workload solutions, and mental health support	No projections	Inadequate training, retention issues, ethical challenges, and policy gaps need to be addressed. Essential measures include specialized geriatric training, ongoing education, mentorship, and mental health support	N/A	Increased awareness of moral distress, workforce burnout, ethical training, retention challenges, collaboration, and mental health support
11	El‐Hneiti, 2018	to identify the predictors of physical and psychological stress among nurses caring for older adults (65 + years) in acute care settings in Jordan	high workload, staff shortages, unsocial hours, emotional burdens, limited training, lack of support, poor work-life balance, and gender disparities, all of which contribute to increased stress	Nurses	Supports stress management, job rotation, organizational climate improvement, professional development, and ethical preparedness as strategies to reduce nurse stress and strengthen workforce capacity	staffing shortages, work overload, inadequate training, and poor organizational climate as key stressors among nurses caring for older adults,	Nurse shortages and the need for better preparation in dementia care	dementia needs trained specialists	No projections	The need for geriatric training and employee development is mentioned	N/A	Stress among nurses and the psychological impact of caring for older adults, with an emphasis on workload, training needs, and organizational support

### Workforce training and professional development

Training and professional development opportunities were commonly cited as essential elements needed to improve workforce planning models [30, 33–35]. Numerous studies also underscored the necessity of more accessible ongoing education and advanced training for the healthcare workforce [29, 30, 32, 33, 35]. For example, in the Iranian context, Nojomi et al. [34] identified that the availability of opportunities for higher educational training of medical specialists could be a significant means of meeting the increasing demand for chronic disease management in older adults. This study initiated an important discourse on how medical education can evolve to adequately equip up-and-coming healthcare practitioners with the skills they need to face real-world adversities in clinical settings. One obstacle to improving workforce capacity was identified as the lack of structured career development and specialized training programs, especially for nurses and caregivers [33, 35] (see Table 3).

### Retention strategies

Workforce retention emerged as a significant concern in most studies, particularly in countries with high turnover rates of healthcare professionals. El-Hneiti et al. and Alameddine et al. [7, 33] both highlighted that poor working conditions, long hours or workloads, limited organizational support, and low salaries were the main contributors to staff dissatisfaction and workforce instability (see Tables 3 and 4). Among these barriers, low salaries were found to be particularly critical in Jordan and Lebanon, where insufficient financial incentives have fueled the migration of skilled healthcare professionals to higher-income countries, worsening shortages in the primary and geriatric care sectors [7, 35] (see Tables 3 and 4). Seven studies recommended strategies to enhance retention, including offering better compensation, increasing opportunities for career advancement, fostering a supportive work environment, improving healthcare infrastructure, addressing staffing shortages, and providing professional development opportunities [7, 27, 30, 32–35] (see Table 4).

**Table 4:** Limitations, recommendations, barriers and future research

Study ID	Author-Year	Study limitations	Policy recommendations	Regulatory barriers	Future research directions
1	Moghadasi, 2022	The study is limited to a single city, Ghaemshahr, Iran. It utilizes a qualitative design	Increase public knowledge about available services, make it easier for older people to access care, and provide a wider range of healthcare services	Problems with the referral process, limited insurance coverage, and poor facility conditions	Should explore ways to improve service awareness, access, elderly care training, and insurance support for older adults
2	Yektatalab, 2012	Qualitative studies’ small sample sizes limit generalizability, and the lack of caregiver training may affect care quality	The study emphasizes the need for specialized caregiver training and improved education on Alzheimer's care for health professionals	Alzheimer’s care in elderly homes is mostly informal, with unwritten routines and limited caregiver training, highlighting gaps in professional development and oversight	To improve the quality of care for Iranian Alzheimer's patients, further studies are recommended to explore various aspects of care and the key themes identified in this research
3	Gharibian Adra, 2015	The study was limited by a small sample size and only one interview with each participant, which may affect the depth and confirmation of the findings	Policies should encourage person-centred care, greater family involvement, and support for spiritual practices in Lebanese care homes	Lebanese care homes lack formal regulations and assessment bodies, leading to inconsistent care quality across facilities	Should expand to more regions, explore spirituality, and use longitudinal designs
4	Nojomi, M., 2023	Small sample size, single university setting, and potential social desirability bias limit the generalizability of the findings	Medical schools should integrate ageism training, strengthen teamwork and communication skills, and improve geriatric care infrastructure	Reveals ageism in healthcare, a shortage of geriatric specialists, and inadequate infrastructure for older adults	Need for initiatives to reduce ageism in healthcare, enhance interprofessional education and improve infrastructure for older adult care
5	Al Shammari, 1997	Conducted in 1997, the study reflects physicians' views and may not capture current practices or perspectives from other stakeholders in elderly home care	Elderly home care in Saudi Arabia should be expanded by integrating PHC teams, with home visits prioritized as a cost-effective alternative to hospital stays	Weak hospital-PHC coordination, underdeveloped elderly care services, and unclear professional roles in home healthcare	Home visit programs for elderly care need stronger training and better logistics
6	Shaheen, 2019	This cross-sectional study captures nurses' perceptions, with limited generalizability and no direct patient outcome measures	Policies should improve work conditions, reduce stress, enhance competence, and ensure staffing to support elderly care	Insufficient staffing, high workload from administrative tasks, and organizational issues impact care quality	Future efforts should improve work conditions, enhance nurse competence, support leadership, and integrate geriatric care into nursing education
7	Wasfi, 2024	The study focussed on two professions, used only Ministry of Health data, and had a small sample size, which limited its generalizability	Policies should expand services regionally, balance the rehabilitation workforce, and include other professions in planning	The study notes a shortage of occupational therapists, unequal workforce distribution, and limited integration of rehabilitation professionals	Future research should assess other rehabilitation professions and explore regional factors affecting workforce distribution
8	Ahmadi, 2015	The study included only Tehran hospitals, excluding private and non-cooperative facilities	The study recommends elderly care departments, national age-friendly programs, and geriatrics training in medical education	There is a shortage of geriatric specialists and no standardized policies or dedicated services for elderly care	Future research should include private hospitals, other regions, and evaluate elderly care services and geriatric training
9	Alameddine, 2015	The study relied on 22 interviews and lacked quantitative data to validate findings	The study suggests mandatory rural service, targeted specialist recruitment, improved salaries and professional development for retention	Barriers include migration to wealthier countries, financial constraints, limited rural specialties, and a lack of incentives for remote work	Future research should assess the long-term impact of retention strategies in rural areas and further explore gender disparities and the integration of mental health and geriatric services into primary healthcare
10	Nasrabadi, 2021	Limitations include small sample size, single location, subjective data, recall bias, cultural limits, no quantitative analysis, policy gaps, and time constraints	The study advocates better staffing, ethical training, emotional support, nurse participation in policies, and enhanced end-of-life care education to ease nurses’ moral distress	Organizational problems, staffing shortages, administrative neglect, and training gaps are discussed	Further qualitative research is needed to understand ICU nurses’ moral distress better and guide effective policymaking
11	El‐Hneiti, 2018	Self-reporting, convenience sampling, and single-city recruitment limit the study’s generalizability	The study recommends rotating nurses, offering stress training, researching interventions, and supporting female staff	The study identifies institutional barriers, such as unsocial work hours, high workloads, and limited organizational support	The study recommends future interventional and qualitative studies to deepen understanding nurse stress, especially when caring for severely ill older adults

### Health financing and sustainability

Another critical point, highlighted in several studies, including five of the eleven studies, was the sustainability of the healthcare funding system. With rising expenditures for healthcare and resources focussed on managing chronic diseases, the sustainability of healthcare in many EMR countries is being challenged. Adra et al. and Alameddine et al. [7, 29] highlighted persistent financial constraints, uneven public sector support, and the need for more robust financial planning to strengthen workforce retention and long-term care services. Al Shammari [27] also emphasized the importance of promoting cost-effective care models, particularly by expanding home healthcare services as an alternative to hospital-based care. Wasfi et al. [26] further underscored the need for targeted investment in the rehabilitation workforce, with a focus on addressing regional disparities and aligning services with national health strategies. Moghadasi et al. [31] also highlighted structural financial barriers, including inadequate insurance coverage and poor healthcare infrastructure, which continue to hinder equitable access to care. These findings demonstrate the urgency of implementing financially sustainable models to ensure equitable, long-term access to quality care for older adults (see Tables 3 and 4). Only one study examined workforce supply and demand quantitatively [26], and none employed formal projection models.

### Barriers to workforce planning

Barriers to workforce planning in the EMR were identified in several studies. Some of the common barriers identified were the lack of healthcare infrastructure and resources, political instability, and the migration of skilled healthcare professionals. For example, Alameddine et al. [7] highlighted major recruitment barriers in Lebanon, including limited incentives for working in rural regions and the migration of the healthcare workforce to rich countries. Moreover, a key challenge identified by Wasfi et al. [26] is the insufficient integration of rehabilitation professionals into the broader healthcare system in Saudi Arabia. In particular, they point to the lack of a standardized workforce framework across regions and the shortage of occupational therapists. Both studies also noted financial constraints and weak regulatory infrastructure as critical obstacles to efficient workforce planning. These findings underscore the need for a systemic approach to address professional shortages, improve the workforce distribution, and enhance recruitment and retention strategies, particularly in underserved and rural regions (see Table 4).

### Limitations and recommendations

Common limitations observed by the authors of this review, including small sample sizes, particularly in qualitative-based research, and limited generalizability due to regional variations in the EMR [7, 28–34]. The studies were also hindered by the lack of longitudinal data, which complicates efforts to evaluate the long-term effects of workforce planning interventions [7, 28–30, 34] (see Tables 3 and 4). Six studies yielded recommendations for strengthening workforce planning in the region. These involved improving data collection on workforce demand, investing in education and training for the healthcare sector, and retaining more healthcare professionals in underserved areas [7, 26, 30, 32, 33, 35] (see Tables 3 and 4). A further common recommendation was to integrate demand-and-supply forecasting models with competency-based approaches to workforce planning as a means to alleviate the impacts of healthcare workforce shortages [7, 26, 30, 35] (see Tables 3 and 4).

## Discussion

This is a mixed-methods systematic review synthesize evidence on healthcare workforce planning for older adults in the EMR. Across studies, recurring including the limited use of workforce projection models, shortage of geriatric and rehabilitation services, inadequate competency-based training, high turnover and workforce migration, and concern regarding financial sustainability. Although the evidence base is limited, and is concentrated in four EMR countries, the findings consistently indicate structural gaps in the workforce's readiness for population aging.

A recurring theme was the need for evidence-based workforce planning models to predict future healthcare needs. As mentioned by Al Shammari [27], there is an ever-increasing demand for these healthcare services, particularly among older adults and those with chronic diseases. Wasfi et al., Al Shammari, and Shaheen et al. [26, 27, 35] suggested that workforce planning should incorporate both demand and supply forecasting approaches to better anticipate future service needs. Despite this recognition, structured supply-and-demand forecasting models were rarely operationalized across the included studies, indicates that workforce planning in EMR remains limited in its application of an integrated, data-driven projection framework. By synthesizing these findings, the present review highlighted a critical evidence gap and underscores the need for contextually adept workforce projection models to strengthen future planning efforts in the region. The identified gaps in training, specialized workforce shortages, and retention challenges provide important contextual parameters that may inform the development of future projection models tailored to the aging population in the EMR.

Workforce planning in the EMR remains an underdeveloped, particularly in an aging population. Limited availability of timely and comprehensive workforce data, alongside weak predictive modeling, constrains accurate forecasting workforce shortages [36]. International efforts, notably those led by the WHO [37], have emphasized the importance of workforce forecasting in responding to demographic aging. Strengthening context sensitive and methodologically robust forecasting models may therefore represent a necessary step toward enhancing healthcare workforce planning capacity within the EMR.

Nine of the eleven studies emphasized importance of workforce training and professional development for the healthcare workforce [7, 27, 28, 30–35]. The absence of a structured career pathways, particularly for nurses and caregivers, was identified as barrier to workforce sustainability [33, 35]. For instance, Nikbakht Nasrabadi et al. [30] highlighted the need for ongoing advanced education for the healthcare workforce, which is especially relevant in light of the projected increase in chronic disease management among older adults.

There is growing recognition for the need for greater investment in geriatric care preparation, with many studies highlighting the lack of formal programs for medical professionals to equip them with the necessary skills to care for an aging population [38]. This gap has been linked to a lack of focussed education and training opportunities and is particularly pronounced in the EMR. These findings suggest that current workforce planning efforts in the region remain insufficiently aligned with a competency-based training framework tailored to the aging population. Workforce development should be grounded in competency-based models that emphasize specialized skills in geriatrics chronic disease management, and palliative.

Workforce retention emerged as a major challenge, especially in countries with high turnover among health professionals. Alameddine et al. and El-Hneiti et al. [7, 33] identified poor working conditions, lower pay, and long hours play significant roles in the dissatisfaction and workforce attrition. These findings that align with evidence from Saudi Arabia, indicating greater reliance on expatriate nurses in urban centers such as Riyadh and Jeddah, where workforce stability is lower [39]. International evidence similarly indicates that improving retention requires addressing both extrinsic factors (e.g., pay, retirement, working hours) and intrinsic motivators such as job enrichment, career advancement, and training opportunity [40].

Given the multifaceted nature of retention a system-level approach integrating financial, regulatory, and professional development reform is required. For example, research suggests that high turnover cannot be rectified with merely financial incentives, although fostering a sustained environment and brokered professional avenues can help [41]. In context of overburdenend health systems, addressing these challenges in the EMR is essential to sustain workforce capacity amid rising demand for older adults care.

Financial sustainability was explicitly or implicitly discussed in four studies, particularly those addressing funding constraints, cost-effectiveness, insurance limitations, and workforce resource allocation [27, 29, 34]. Increased pressure on health budgets reflects growing demands for long-term, chronic disease management and rehabilitation services. The increasing load of chronic diseases that involve long-term management and specialized care further compounds this challenge [26, 30]. Despite acknowledging these financial pressures, none of the studies conducted a formal cost-effectiveness or economic evaluation analysis of proposed workforce strategies.

One study highlighted the need to strengthen rehabilitation services to address regional gaps and better align services with needs of an aging population [26, 30], but the economic aspect of reforms was not assessed in a systematic manner. Expanding the health workforce require sustained investment in training, employment conditions, and regulatory system [37]. In the absence of economic modeling, policymakers face limited evidence to guide decisions on the long-term cost-effectiveness and sustainability. Meanwhile, multidisciplinary workforce approaches have been proposed to strengthen chronic disease management and improve alignment between care delivery and healthcare utilization demands [42]. Workforce planning should therefore be aligned with broader universal health coverage and health financing reform. Future research should incorporate an economic evaluation model to assess both short-term fiscal requirements and long-term economic cost implications. Besides forecasting and funding disparities, structural impediments still exist in the region. Lack of political stability, poor governance, disjointed infrastructure, and migration stifle coordinated workforce planning. Wasfi et al. [26] identified a lack of proper integration of rehabilitation professionals into large health systems, with Alameddine et al. [7] defining the problem of recruitment in rural and underserved regions. As the aging population increases, there is an immediate need to incorporate rehabilitation specialists, such as occupational therapists, into the health workforce to meet the demand for chronic disease management and long-term care [43]. Kattan and Al-Hanawi [39] reported that despite efforts to increase the proportion of Saudi nurses through Saudization, the private sector continues to rely significantly on expatriates, contributing to regional workforce imbalance. This results in marked inequalities in the distribution of nurses across regions, with underserved regions lagging behind. Such findings are in line with global studies that emphasize the adverse effects of the migration of health professionals on the health workforce in developing countries [44, 45]. Addressing these barriers requires regulatory reform, strengthening health workforce data systems and improving intersectoral coordination [46].

### Implications for policy across EMR contexts

The EMR is heterogeneous, and policy responses should therefore be context-specific.

- In Gulf Cooperation Council (GCC) countries, investment in workforce modeling, geriatric subspecialization, and localization strategies may reduce reliance on expatriate labor and improve stability.
- In middle-income EMR countries, priorities include strengthening primary care, integrating geriatric competencies, and enhancing retention through structured career development pathways.
- In fragile or conflict-affected settings, workforce resilience, task shifting, and regional collaboration mechanisms may offer pragmatic short-term solutions.

In this context, workforce planning should prioritize long-term strategic realignment aligned with demographic and epidemiological transition.

## Limitations and recommendations for future research

This review has several limitations. Only 11 studies from four EMR countries met the inclusion criteria, limits the generalizability. Most of the included studies were cross-sectional or small-scale qualitative, restricting causal inference and long-term evaluation of workforce intervention. Only one study addressed workforce supply and demand quantitatively, and none used formal workforce projection models, limited economic analysis and limiting assessment of financial impact. Inclusion of English-language publications only may have introduced language bias. Heterogeneity in study design, setting and outcome measures further restricted direct comparison. These limitations highlight the need for multi-country longitudinal and economically informed workforce planning research in aging EMR contexts.

Al Shammari (1997) was included in accordance with the review’s predetermined eligibility criteria. However, its findings should be interpreted as a historical insight informing the evolution of workforce planning frameworks rather than as direct guidance for the contemporary health system, given the temporal gap between publication and current system realities.

Future research should prioritize evaluating the effectiveness of workforce planning models, including demand and supply forecasting, competency-based training approaches, and retention strategies. It is also essential to examine the role of health financing mechanisms in ensuring the long-term sustainability of workforce development programmes, particularly in resource-constrained settings.

## Conclusion

This systematic review highlights significant gaps in health workforce planning of elderly in the WHO EMR. The issues of workforce shortages, migration, and the lack of training are well known, but aging-specific projection models and a combined policy framework are scarce. Financial sustainability factors are often recognized but not formalized in economic analysis. These gaps also need to be addressed by creating data-driven forecasting models, competency-based training plans, context-sensitive retention policies, and workforce plans aligned with health financing reforms. To make the EMR health systems more responsive to the increasing and sophisticated demands of aging populations, it will be necessary to strengthen these components.

## Supplementary Information

Supplementary material 1.

## Data Availability

Not applicable.
