# Supplementary figures and images for: Health workforce planning models for older adults in the WHO Eastern Mediterranean Region: a mixed methods systematic review
Source: Hum Resour Health. 2026 May 15;24:38. doi: 10.1186/s12960-026-01077-9 (PMC13348860; doi:10.1186/s12960-026-01077-9)

# **Supplement/Annexures**

**The Systematic Review Registration**
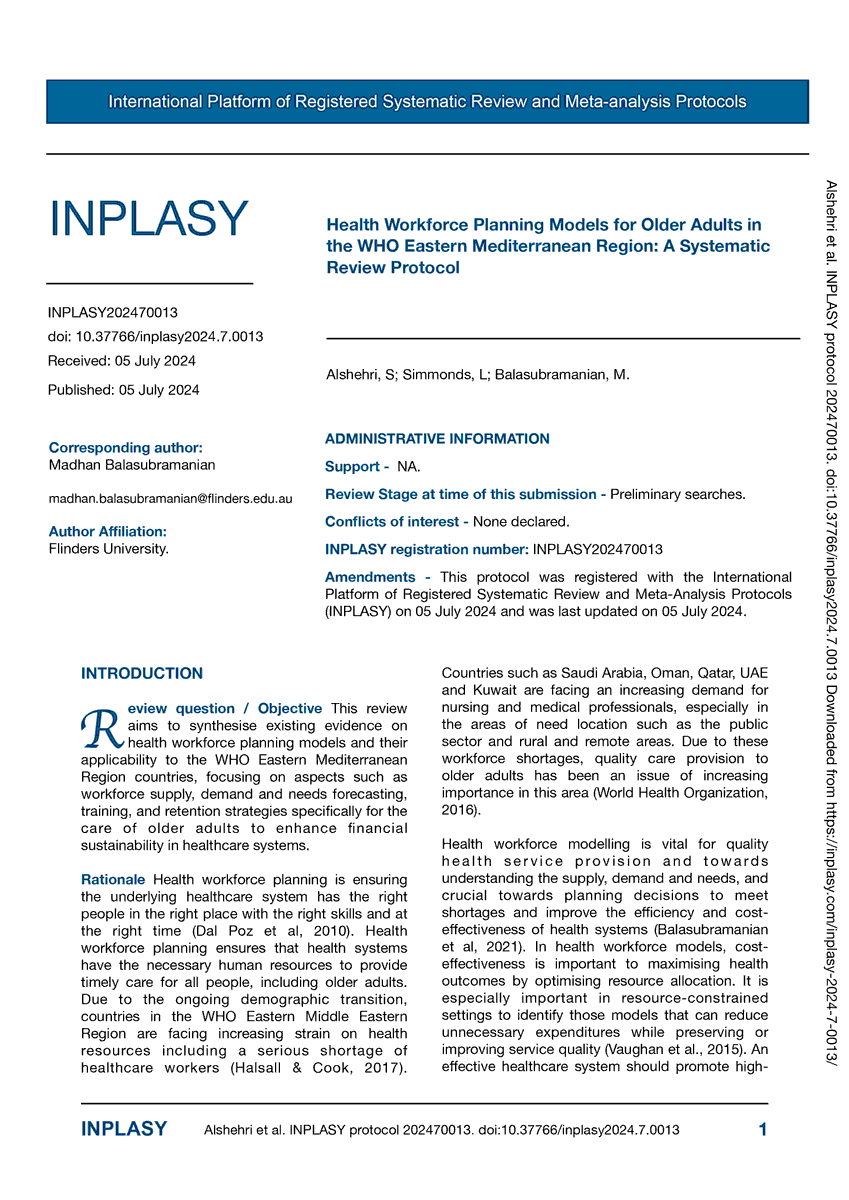

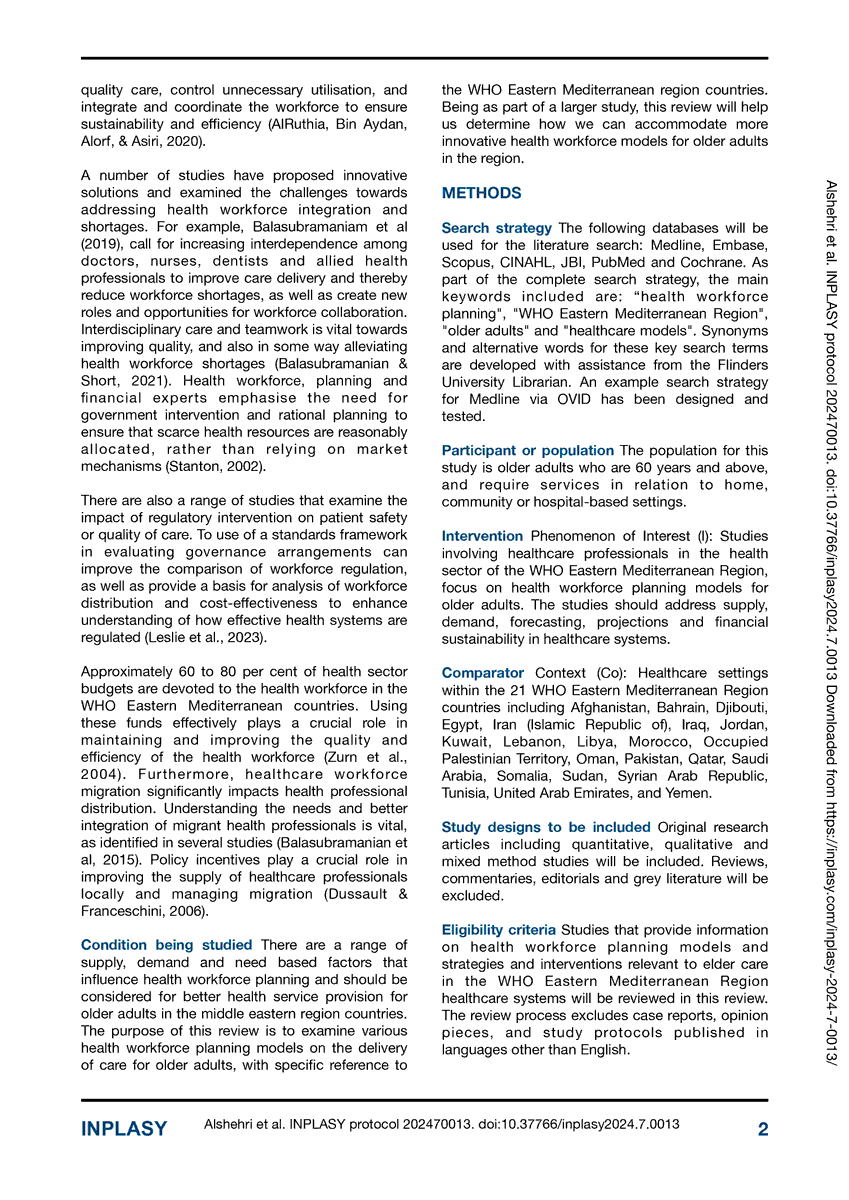

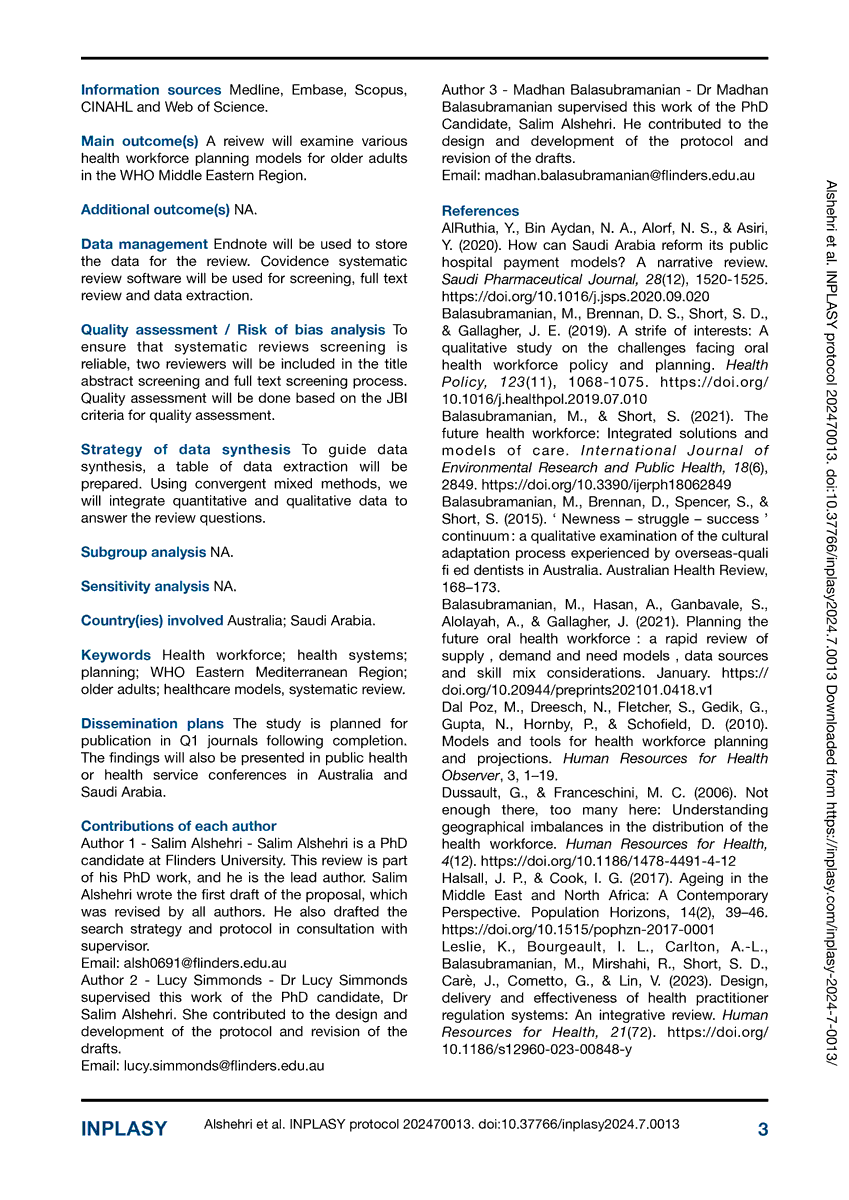

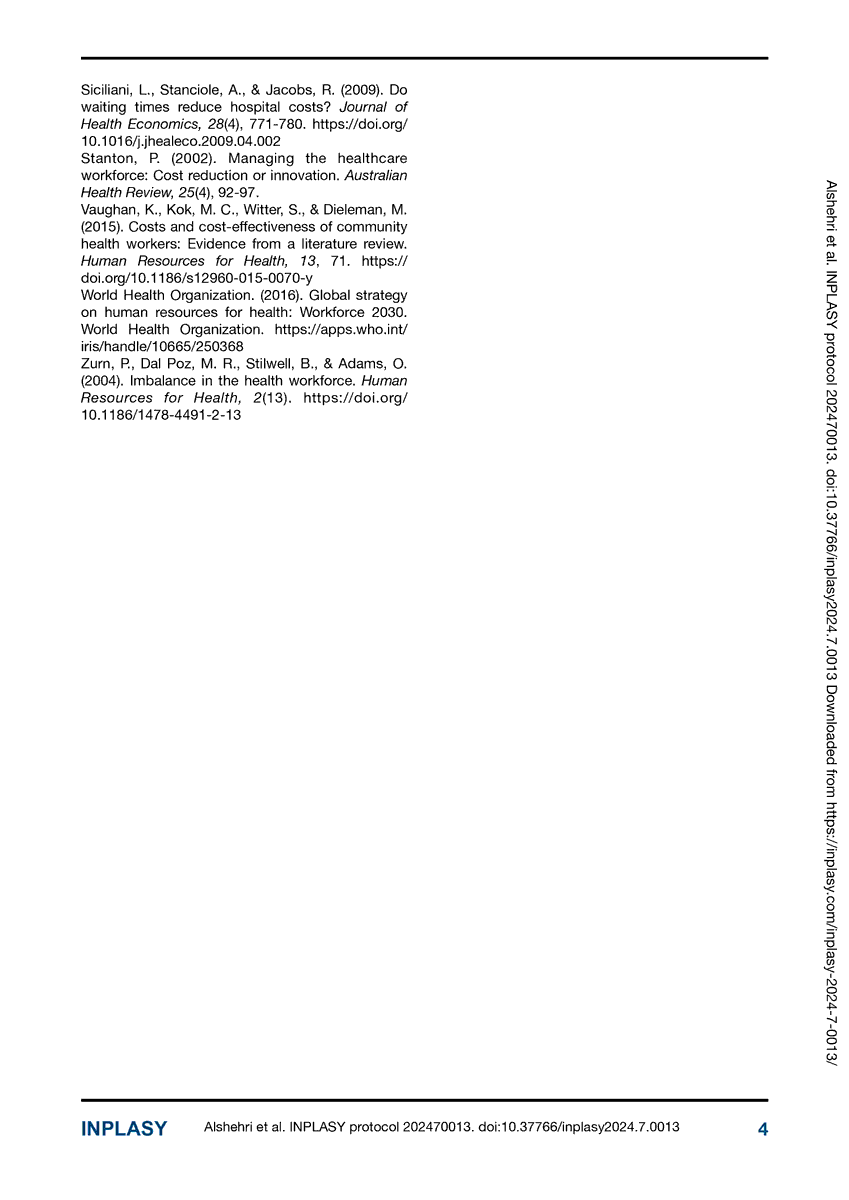

Supplement: Supplementary file 1 — Supplementary material 1. [file 12960_2026_1077_MOESM1_ESM.docx]
